# Optimization of Drying Parameters for Total Phenolic Content of Papaya Using Response Surface Methodology

**DOI:** 10.1155/2022/4819725

**Published:** 2022-12-22

**Authors:** Ferdusee Akter, Shireen Akther, Afroza Sultana, Md. Motiur Rahman, Ujjwal Kumar Deb

**Affiliations:** ^1^Department of Mathematics, Chittagong University of Engineering & Technology, Chattogram-4349, Bangladesh; ^2^Department of Physical and Mathematical Sciences, Chittagong Veterinary and Animal Sciences University, Chattogram-4225, Bangladesh; ^3^Department of Food Processing and Engineering, Chittagong Veterinary and Animal Sciences University, Chattogram-4225, Bangladesh

## Abstract

An optimum condition of the drying process can minimize nutrient losses and maximize the shelf life of food products. Thus, this study is aimed at developing an optimized system for the process conditions to determine the total phenolic content (TPC) of oven-dried papaya slices. The response surface method and central composite design were used to design the experiment, and it was found that the drying conditions had a significant impact on the total phenolic content of papaya slices. TPC was determined in relation to their interactions with the independent variables that include time, temperature, sample thickness, and stage of ripeness. The optimum drying conditions are those with the maximum content of TPC. In order to fit the experimental data, a quadratic polynomial model is created for the output variable, and an analysis of variance is carried out to determine whether or not the model is compatible to determine the optimal drying conditions. Time (10 h), temperature (62.02°C), thickness (9.75 mm) and stages (ripe) were found to be the optimal drying conditions. It was found that temperature had more effect on the amount of TPC than other factors. The numerical findings showed a good agreement with experimental data, with *R*^2^ = 0.9237. It is hoped that the findings will make a contribution to the process of drying food.

## 1. Introduction

Postharvest losses of fruits are a big issue all over the world. After being harvested, a significant number of fruits are lost due to deterioration. Depending on the country and commodity, this loss might vary from 10% to 50% [1–4]. Moreover, it has been estimated that 20–25% of fruits and vegetables throughout the world are lost to fungal and bacterial diseases after harvest. Furthermore, it is common in underdeveloped nations for postharvest losses to be more severe because of limited storage and transportation facilities. In Bangladesh, a wide range of nutrient-dense and mouthwatering fruits are grown because of the country's tropical and subtropical climate. There is still a large amount of produced yield that never reaches the end user owing to postharvest losses [5]. There is now a demand to reduce the postharvest loss by processing. Because of this, the product needs the right postharvest processing technology to make it last longer.

Papaya (*Carica papaya L.*) is a lovely and delicious tropical fruit with a distinct pleasant aroma. It is one of the most affordable and nutrient-dense fruits, accessible all year round. In Bangladesh, green papaya is a popular vegetable while ripe papaya is a popular fruit, loved by people of all ages. It has a buttery consistency and a soft texture [6]. Christopher Columbus termed this lovely fruit “The Fruit of Angels.” This fruit is also known as the “fruit of long life”, and the plant is called the “tree of health”. The ripening process enhances the flavour and sweetness of the fruit. Overripe fruit, on the other hand, begins to degrade swiftly in quality [7]. When compared to other foods, papaya contains a greater number of nutrients per calorie and hence may be called a nutrient-dense food. Additionally, it has high levels of potential antioxidants and bioactive compounds.

The high water content in fruits makes them more perishable foods. Drying is one of the most common ways to preserve fruits with minimum nutrient loss. Drying is a method used to preserve food, which involves the simultaneous transfer of heat, mass, and momentum to remove moisture. It prevents the growth of bacteria and other unwanted organisms and increases the shelf life of the food product. Moreover, drying reduces the cost of packaging, storing, and transporting food by lowering the weight and volume of the product [8]. More than 85% of the dryers used in the industry today are of the convection type and use hot air for drying, making this method the most cost-effective drying method [8, 9]. Both the thermophysical properties of the food and the drying conditions affect the drying process. The price of the product as a consequence of its quality must be taken into account when choosing the drying conditions [10]. TPC is a bioactive compound in papaya with a unique structure and a set of potential health advantages and it may reduce due to drying. TPC may improve health through its physiological effects. Furthermore, it has a key role in protecting the human body from chronic disease [11]. Researchers are looking into its potential use in the treatment and prevention of cancer, heart disease, and other illnesses. Therefore, it is crucial to choose the best drying conditions to ensure that the nutritional values are preserved.

Technology development cannot proceed without first employing optimization in order to achieve the necessary quality characteristics in the final product. The design of experiments (DoE) may be seen as a strategy for optimizing the amount of necessary testing required to identify the specific factors that affect the process. The response surface methodology (RSM), is a statistical tool that is frequently implemented in optimization research to solve problems in scientific and engineering research [12]. It is a method for designing, enhancing, and optimizing processes by employing mathematical and statistical techniques, and it is also used to identify the interactions between various influencing elements and variables. It optimizes a dependent parameter as the output variable. The RSM approach is ideal for fitting a quadratic surface and aims at the optimization of process parameters with a small number of tests as well as the analysis of parameter interactions. Many researchers have found success with the statistical approach for optimization known as the response surface methodology [12–15].

Since no studies have been conducted on the optimal drying conditions for papaya at different ripening stages, this study is aimed at optimizing the hot air drying process. Total phenolic content of dried papaya slices was investigated by testing the impact of drying time, drying air temperature, sample thickness, and ripening stage using a response surface approach.

## 2. Materials and Methods

### 2.1. Sample Preparation

At first, papayas in various stages of ripeness (green, semiripe, and ripe) were procured from the local market (Jhautala Bazar, Pahartali) of Chattogram. After collecting the fruits, they were properly cleansed to remove any dirt, ferns, or other debris that may have accumulated. Then, the fruits were peeled and sliced, and dried them at temperatures of 60°C, 70°C, and 80°C in a hot air oven (Model: redLINE RE 53).

When the drying process was finished, the samples were put into desiccators for 30 minutes to cool down. The dried samples were ground and kept in airtight containers for further analysis. The tests were carried out in triplicate, and the results were reported as percentages based on a dried sample (% db).

### 2.2. Preparation of the Extract

Extracts were prepared using a modified version of the procedure reported by Unal et al. [16]. Absolute ethanol was added to the papaya powder samples in their individual beakers, and the mixture was allowed to shake for 72 hours at room temperature. The remaining residue was strained to separate the solvent. After the initial extraction, the filtrate was collected and kept at room temperature while the extraction process was repeated twice with a new solvent. Crude extracts were obtained by combining all filtrates and evaporating them at 60°C in a rotary evaporator (Heidolph TM Hei-VAP Digital Model). The crude extracts were weighed and stored at 4 °C until further analysis.

### 2.3. Determination of Total Phenolic Content (TPC)

The TPC of the extracts from the papaya samples was calculated using the published technique with some minor adjustments [17]. Standard solutions of gallic acid (Sigma, USA) (0.02, 0.04, 0.06, 0.08, and 0.10 mg/mL) and stock solutions of extracts (1 mg/mL) were made. A cuvette was pipetted with either an extract or a standard solution of Gallic acid (0.3 mL). Then, 1.5 mL of diluted Folin-Ciocalteu reagent was added and stirred in. After waiting 3 minutes, 1.5 mL of sodium carbonate (75 g/L) solution was added, and the mixture was left for another 60 minutes. The absorbance was measured by UV spectrophotometer (Model: UV-2600) at 765 nm. The ethanol was used as the blank. Gallic acid equivalent (GAE) per gram of extract (mg GAE/g) was used to measure TPC.

### 2.4. Experimental Design and Data Collection

A standard response surface design, named the central composite design (CCD), was used to investigate the parameters of the quality drying of papaya slices. RSM may also be used to figure out how response and control variables are related [18, 19]. The Trial version of Minitab 21.2.0.0 software was used to design the experiment and analysis of the results.

The conditions of the experiment were chosen at a range of different levels according to preliminary testing and literature reviews [19–22]. Drying time, *X*_1_ (8h, 9 h, and 10 h), drying temperature, *X*_2_ (60°C, 70°C, and 80°C), thickness, *X*_3_ (5, 7.5, and 10 mm), stage of ripening, *X*_4_ (green, semiripe, and ripe) were the independent factors in this investigation. To study the individual and interactive effects of drying time, drying air temperature, sample thickness, and ripening stage on the quality properties of dried papaya, a 4 × 3 face centered CCD of RSM was adopted. The output variable was TPC. The design involved 60 runs with 59 degrees of freedom.

The control factors with levels are represented in Table 1.  Table 2 lists the 60 CCD experiments that were carried out as part of the optimization process. Each RSM output variable and the experimental parameters are linked mathematically by a nonlinear polynomial equation with squared terms, two-factor interaction terms, linear terms, and a constant term. The equation can be represented [12] as follows:

(1) $$\begin{array}{c} Y=\beta_{0}+\overset{m}{\underset{i=1}{\sum }}\beta_{i}X_{i}+\overset{m}{\underset{i=1}{\sum }}\beta_{ii}X_{i}^{2}+\overset{m}{\underset{i=1}{\sum }}\overset{m}{\underset{j=i+1}{\sum }}\beta_{ij}X_{i}X_{j}+\varepsilon , \end{array}$$

where *Y* is the response variable, *β*_0_ is the constant; *β*_i_, *β*_*ii*_ and *β*_*ij*_ are the coefficients of linear terms, quadratic terms, and interaction terms, respectively; *X*_i_ and *X*_j_ are the independent variables; m is the number of variables; *ε* represents the random error of the model. The two-way and quadratic interactions are shown by the variables *X*_*i*_*X*_*j*_ and *X*_i_^2^, respectively.

The selection of significant interactions between variables over the response determines the probability of a maximum response. The analysis of variance (ANOVA) was used to figure out how many linear, quadratic, and two-way interaction factors affected the results and how their regression coefficients changed. After the optimization process, the best settings for the variables were found. These settings were then used in a lab experiment, and the expected and actual response values were compared.

## 3. Results and Discussions

### 3.1. Statistical Analysis of the Results of Model Fitting

The results of the experiments on the response variable according to the various drying conditions are presented in Table 2. The initial values of TPC were found 18.87 ± 0.07 mg/100 g, 15.22 ± 0.08 mg/100 g and 13.39 ± 0.11 mg/100 g for green, semiripe and ripe papaya samples, respectively. The evaluation of the statistical data was carried out with the trial version of the statistical software Minitab 21.2.0.0. To test the reliability and fitness of the model, an ANOVA was carried out [19, 21]. An average value from each trial was used to fit a second-order polynomial model, yielding the regression equations. The performance of a model in making predictions can be measured using its predicted *R*^2^. To figure out how well the model worked, the mean square, sum of squares, degree of freedom (DoF), *P* value, and *F* value were each calculated. According to the results of the variance analysis, an *F* value that is more than 2.6 suggests a more accurate estimation of the parameters. Additionally, if the *P* value is less than 5%, the statistical model is accepted.

The results of the ANOVA on TPC content are presented in Table 3. A *P* value for the model below 0.05 implies that there is a statistical significance between the terms in the model. Since the *F* value of the model is 29.93, it can be concluded that it is statistically significant. The terms with *P* values below 0.05 are considered significant. The predicted *R*^2^ value of 0.8394 and the adjusted *R*^2^ value of 0.8929 are quite close to one another. The *R*^2^ value for the response variable was 0.9237, indicating that 92.37% of the total variance was well explained by the model. Since the adequate precision value of 10.92 is more than 4, we may conclude that this response was more accurate and trustworthy. The lack of fit is insignificant, which also validates the accuracy of the model. As a result, it is possible to optimize this response variable by applying the model.

**Figure 1:**
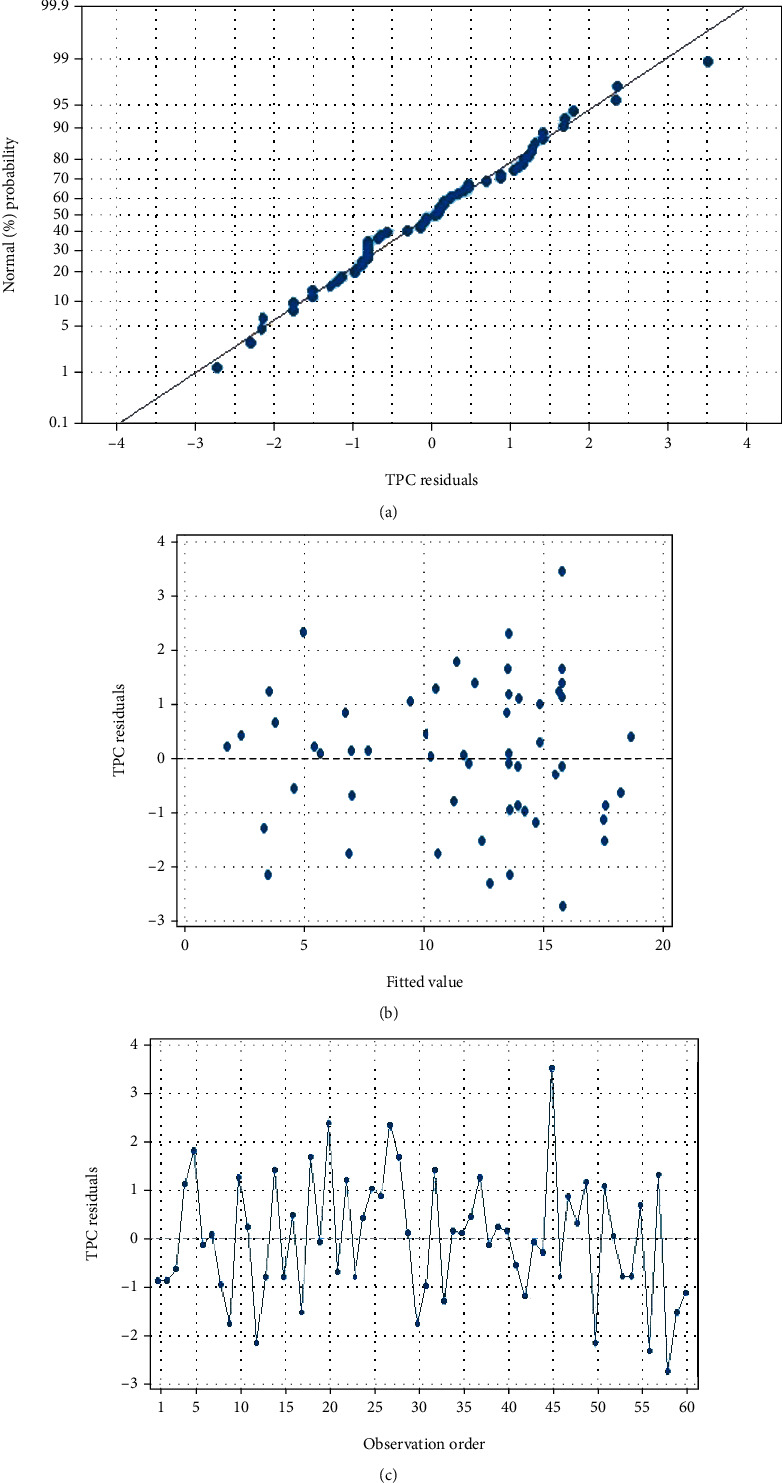
(a) Normal Probability vs residuals, (b) residuals vs fitted value, and (c) residuals vs. observation order for TPC.

Figure 1 illustrates the normal probability plot of the residuals (Figure 1(a)), the random distribution of the residuals (Figure 1(b)), and the residuals versus observation order for the TPC (Figure 1(c)). It has been seen that the values are rather close to a straight line, which demonstrates that the model is accurate. The distribution of residuals has a range of values from -3 to 4, with 45, 56, and 58 having observation orders with the highest residuals. So, the model gives a good explanation of how the analysis of variance of the response works [19].

The TPC content for different papaya samples can be expressed by the following equations:

(2) $$\begin{array}{c} {\text{TPC}}_{\text{green}}=-79.0-16.64X_{1}+4.96X_{2}+0.54X_{3}+0.84{X_{1}}^{2}-0.0368{X_{2}}^{2}-0.2066{X_{3}}^{2}-0.0127X_{1}X_{2}+0.413X_{1}X_{3}-0.0132X_{2}X_{3},\\ {\text{TPC}}_{\text{semi ripe}}=-63.4-17.80X_{1}+4.94X_{2}+0.36X_{3}+0.84{X_{1}}^{2}-0.0368{X_{2}}^{2}-0.2066{X_{3}}^{2}-0.0127X_{1}X_{2}+0.413X_{1}X_{3}-0.0132X_{2}X_{3},\\ {\text{TPC}}_{\text{ripe}}=-51.4-18.26X_{1}+4.82X_{2}+0.71X_{3}+0.84{X_{1}}^{2}-0.0368{X_{2}}^{2}-0.2066{X_{3}}^{2}-0.0127X_{1}X_{2}+0.413X_{1}X_{3}-0.0132X_{2}X_{3}. \end{array}$$

### 3.2. Effect of Drying Conditions on TPC

**Figure 2:**
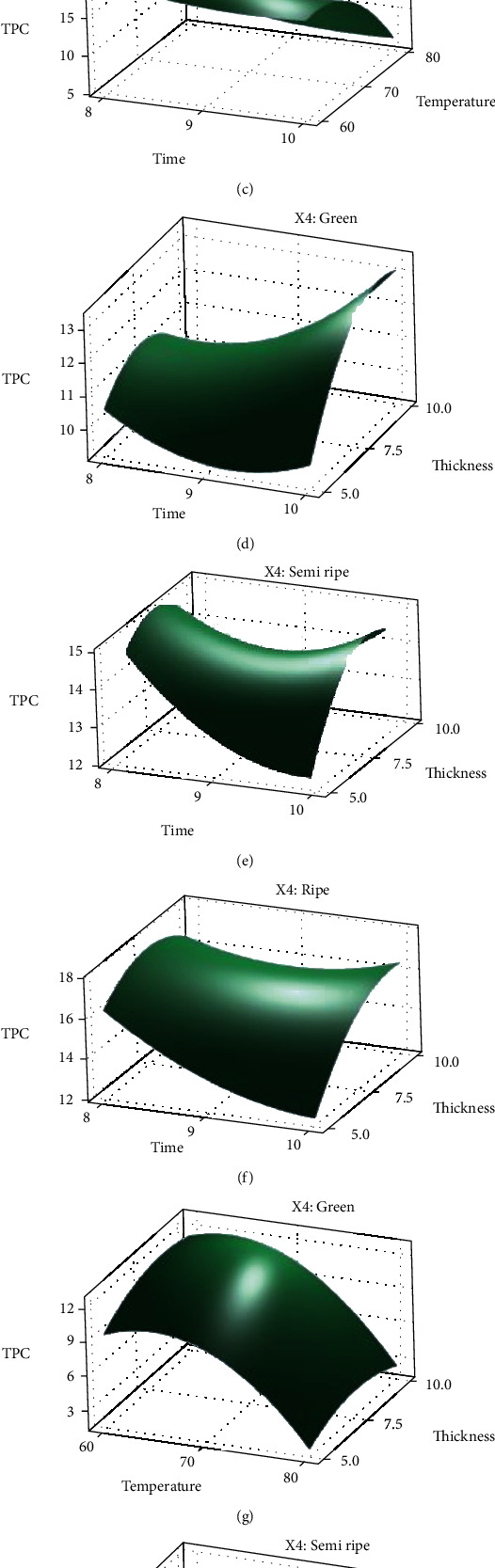
Surface plots of TPC as a function of time, temperature, and thickness for green, semiripe, and ripe papaya samples.

The total phenolic content of papaya is depicted in Figure 2. Response surface plots were generated for the fitted model as a function of two independent variables, while maintaining the third variable at its centre, in order to highlight the cumulative impacts of the variables on the responses. These plots can be found in Figures 2(a)–2(i). The relationships between many different aspects of the experiment and the results are illustrated in a three-dimensional graph.

Increasing the drying temperature led to a substantial drop in TPC for papaya, while a slower decline over time was insignificant. TPC, however, increased noticeably for all samples as their thickness was increased (Table 2 and Figure 2). As shown by the model's high coefficient of determination (*R*^2^ = 0.9237), predictions of experimental and simulated values of TPC are very consistent with one another. According to the surface plots, the TPC content of ripe papaya is the highest and that of green papaya is the lowest.

The findings are correlated with some other published studies [23, 24]. They also found that the thickness of the sample has a considerable impact on the drying process of potato slices and okra slices. The outcomes are also comparable when tomato slices were dried using vacuum-drying method by Abano et al. [22]. Researchers have revealed that when comparing the TPC of dried papaya with that of fresh papaya, the reduction of TPC varies from 7% to 69% [25, 26]. The reduction of TPC in papaya was less in convective drying of papaya rather other drying processes [27].

**Figure 3:**
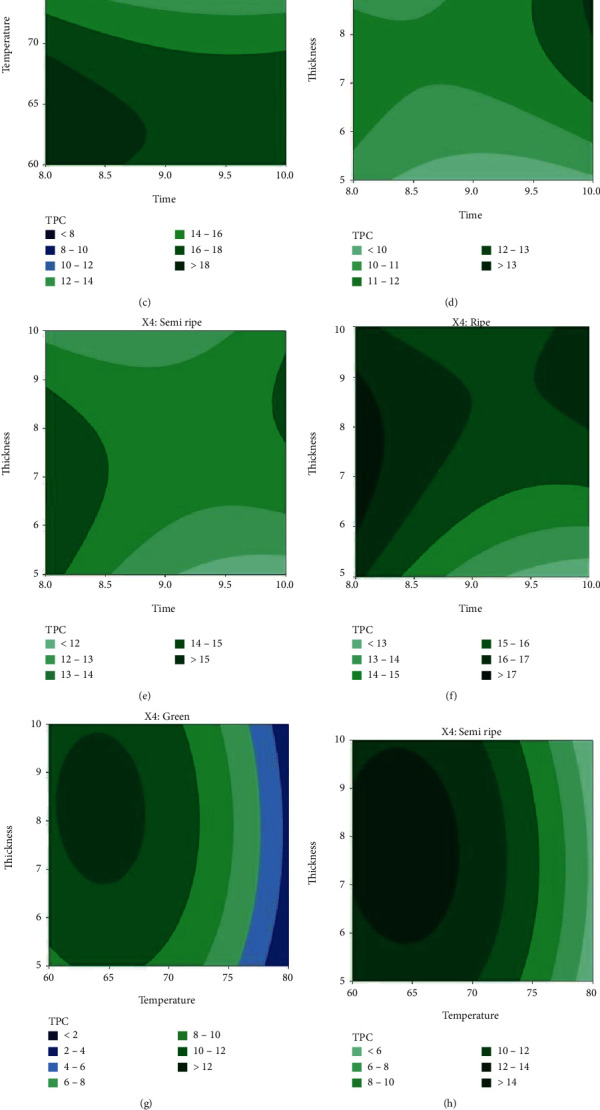
Contour plots of TPC as a function of time, temperature, and thickness for green, semiripe, and ripe papaya samples.

The TPC of three different phases of papaya is shown on two-dimensional graphs in Figures 3(a)–3(i) as a function of drying time, drying air temperature, and sample thickness. TPC levels are seen to change a little for temperature below 70°C and drop gradually when temperatures rise above 70°C. The results show that in all stages of papaya, the highest TPC levels were reported at temperatures between 60°C and 70°C (Figures 2(a)–2(c) and 3(a)–3(c)). Additionally, temperature has a substantially higher *F* value than the other parameters. Therefore, it is evident that temperature has a more significant influence than others in determining the amount of TPC in the drying process. A study found that papaya that had been osmotically pretreated retained most of its bioactive compounds at a temperature of 70°C [28]. Similarly, the grape pomace retained most of its bioactive compounds at a temperature of 60°C [29].

Figures 2(d)–2(f) and 3(d)–3(f) represent the combined effect of time and thickness on the total phenolic content of papaya. It is seen that the amount of TPC has been increased with the increase of time and thickness and the highest value is obtained near 9 mm thickness of the samples.

The well-depicted combined effect of temperature and thickness on the total phenolic content of papaya are shown in Figures 2(g)–2(i) and 3(g)–3(i). According to the figures, the highest value of TPC is found when the temperature is kept below 70 °C and thickness is between 7 mm and 10 mm.

### 3.3. Numerical Optimization and Verification of the Model

**Figure 4:**
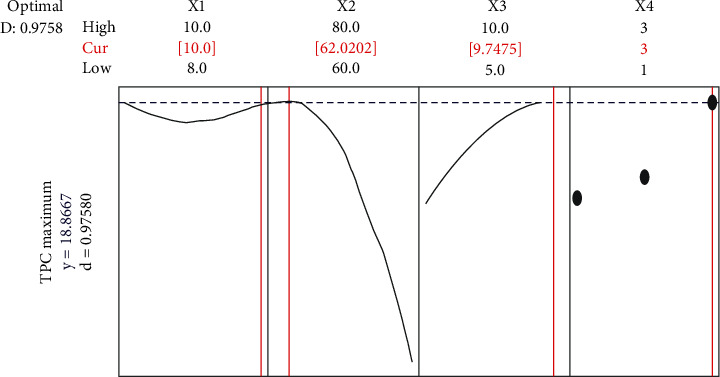
Desirability function with the optimum drying conditions.

The optimization was carried out using Minitab to find the optimum drying conditions for papaya that produced the highest TPC. Through numerical optimization, the ideal drying condition that maximizes the desirable function is shown in Figure 4. When compared to the original values of TPC, similar findings for TPC for papaya drying have been found [27, 28].

Finally, drying experiments were carried out under the ideal drying conditions of 10 hours, 62°C, and a thickness of 9.75 mm for ripe papaya. The response value that was achieved is shown in Table 4 below. The predicted value of the response is in close agreement with the experimental results. These results verify that the RSM model correctly predicted the outcome.

## 4. Conclusions

An experimental design was developed to model and analyse the impact of drying conditions on the total phenolic content of papaya slices using RSM. The results showed that the total phenolic content dropped considerably with an increase in temperature and insignificantly with time. The value of TPC, on the other hand, increased noticeably larger with increasing sample thickness. It was observed that the amount of TPC was significantly more affected by temperature than by any of the other factors. The experimental data is fit to a quadratic model. The optimum value of TPC was obtained at drying conditions of 10 hours of time, 62.02°C of temperature, and 9.75 mm of thickness for ripe papaya. The predicted response value agrees quite well with the experimental findings, with an error of 6.1% and a desirability of 0.9758. These findings provide more evidence that the RSM model made an accurate prediction of the outcome. It is expected that this research will contribute to the food drying process. Moreover, the optimization process can be used to determine the physical and chemical properties of dried papaya.

## Figures and Tables

**Table 1:** Level of control variables.

Variables/control factors	Coded symbols	Levels		
		Level 1 Low, (-1)	Level 2 Mid, (0)	Level 3 High, (1)
Time (h)	*X*_1_	8	9	10
Temperature (°C)	*X*_2_	60	70	80
Thickness (mm)	*X*_3_	5	7.5	10
Stages of ripeness	*X*_4_	Green	Semiripe	Ripe

**Table 2:** Central composite design and results for the response variable (TPC) following the drying process.

Run no.	Time *X*_1_	Temperature *X*_2_	Thickness *X*_3_	Stage *X*_4_	TPC (db) (mg/100 g)
1	10	70	7.5	Semiripe	13.04
2	8	60	5	Ripe	16.78
3	8	60	10	Ripe	17.65
4	10	60	10	Green	15.13
5	8	70	7.5	Green	13.22
6	9	70	7.5	Ripe	15.65
7	9	60	7.5	Green	11.74
8	9	70	7.5	Semiripe	12.61
9	10	80	10	Ripe	5.04
10	10	70	7.5	Ripe	16.96
11	8	80	10	Green	1.91
12	10	80	5	Ripe	1.22
13	9	70	7.5	Green	10.43
14	9	70	5	Semiripe	13.57
15	9	70	7.5	Green	10.43
16	10	60	5	Green	10.52
17	9	60	7.5	Ripe	16.09
18	9	70	7.5	Ripe	17.48
19	9	70	7.5	Semiripe	13.48
20	10	80	10	Green	7.22
21	8	80	5	Semiripe	6.26
22	9	70	7.5	Semiripe	14.78
23	9	70	7.5	Green	10.43
24	10	60	10	Ripe	19.13
25	8	70	7.5	Semiripe	15.91
26	9	70	5	Ripe	14.35
27	9	70	7.5	Semiripe	15.91
28	8	60	10	Semiripe	15.22
29	9	80	7.5	Semiripe	5.65
30	9	70	10	Green	8.78
31	9	60	7.5	Semiripe	13.22
32	9	70	7.5	Ripe	17.22
33	8	80	5	Green	1.913
34	8	80	10	Ripe	7.043
35	9	70	7.5	Semiripe	13.65
36	10	80	5	Green	2.70
37	9	80	7.5	Green	4.70
38	10	60	5	Ripe	13.83
39	10	80	10	Semiripe	5.57
40	8	80	5	Ripe	7.74
41	8	80	10	Semiripe	3.91
42	8	60	5	Semiripe	13.48
43	10	60	5	Semiripe	11.83
44	9	70	10	Ripe	15.22
45	9	70	7.5	Ripe	19.30
46	9	70	7.5	Green	10.43
47	9	80	7.5	Ripe	7.48
48	10	60	10	Semiripe	15.22
49	9	70	7.5	Ripe	16.96
50	9	70	7.5	Semiripe	11.39
51	9	70	5	Green	10.43
52	8	60	10	Green	10.35
53	9	70	7.5	Green	10.43
54	9	70	7.5	Green	10.43
55	10	80	5	Semiripe	4.35
56	10	70	7.5	Green	10.43
57	8	60	5	Green	11.83
58	9	70	7.5	Ripe	13.05
59	9	70	10	Semiripe	10.87
60	8	70	7.5	Ripe	16.43

**Table 3:** ANOVA for TPC.

Source	DoF	Sum of squares	Mean squares	*F* value	*P* value
Model	17	1173.62	69.037	29.93	<0.001
Linear	5	866.55	173.310	75.13	<0.001
*X*_1_	1	1.86	1.864	0.81	0.374
*X*_2_	1	646.86	646.857	280.40	<0.001
*X*_3_	1	10.18	10.183	4.41	0.042
*X*_4_	2	207.64	103.822	45.01	<0.001
Square	3	249.43	83.144	36.04	<0.001
*X*_1_*X*_1_	1	5.81	5.811	2.52	0.120
*X*_2_*X*_2_	1	111.88	111.875	48.50	<0.001
*X*_3_*X*_3_	1	13.75	13.754	5.96	0.019
2-way interaction	9	57.64	6.405	2.78	0.012
*X*_1_*X*_2_	1	0.39	0.386	0.17	0.685
*X*_1_*X*_3_	1	25.59	25.591	11.09	0.002
*X*_1_*X*_4_	2	14.01	7.004	3.04	0.059
*X*_2_*X*_3_	1	2.61	2.609	1.13	0.294
*X*_2_*X*_4_	2	11.11	5.555	2.41	0.102
*X*_3_*X*_4_	2	3.94	1.969	0.85	0.433
Error	42	96.89	2.307		
Lack-of-fit	27	62.13	2.301	0.99	0.523
Pure error	15	34.76	2.317		
Total	59	1270.51			
*R* ^2^: 0.9237	Adjusted *R*^2^: 0.8929			Predicted *R*^2^: 0.8394	
Adequate precision: 10.92					

**Table 4:** Optimum conditions for the drying process.

*X*_1_ (h)	*X*_2_ (°C)	*X*_3_ (mm)	*X*_4_	TPC (predicted) (mg/100 g)	TPC (experimental) (mg/100 g)	Composite desirability
10	62.02	9.75	Ripe	18.8667	20.09 ± 0.04	0.9758

## Data Availability

All data used during the current study are available from the corresponding author upon request.
